# The Role of Skeletal Muscle in Amyotrophic Lateral Sclerosis: State of the Art 2025

**DOI:** 10.3390/muscles4030022

**Published:** 2025-07-09

**Authors:** Elisa Duranti

**Affiliations:** School of Medicine and Surgery, University of Milano-Bicocca, 20900 Monza, Italy; e.duranti@campus.unimib.it

**Keywords:** muscle, neurodegenerative disease, amyotrophic lateral sclerosis

## Abstract

Amyotrophic lateral sclerosis (ALS) is a progressive disease that degeneratively damages both upper and lower motor neurons, eventually resulting in muscular paralysis and death. Although ALS is broad in scope and commonly thought of as a motor neuron disease, more active research sheds light on the that role skeletal muscle plays in the development and progression of the disease. Muscle tissue in ALS patients and in animal models demonstrates severe regenerative deficits, including impaired myogenesis and impaired myoblast fusion. In ALS, muscle stem cells, known as satellite cells, show poor performance in activation, proliferation, and differentiation and thus contribute to ALS muscle wasting. Moreover, the pathological tissue environment that inhibits myoblast fusion is made up of proinflammatory cytokines, oxidative stress, and a lack of trophic signals from the neuromuscular junction, which greatly disrupts homeostatic regulation. It is likely that skeletal muscle is instead a dynamic player, fueling neuromuscular degeneration as opposed to a passive responder to denervation. One must appreciate the cellular and molecular changes that complicate muscle regeneration in ALS for effective treatment to be developed, permitting simultaneous interventions with both muscle and neurons.

## 1. Introduction

Amyotrophic Lateral Sclerosis (ALS) is a motor neuron disease that leads to the degeneration of upper motor neurons located in the brain and lower motor neurons situated in the spinal cord and brainstem. These neurons help in voluntary muscle movement. Damage to the neurons results in muscle weakness, atrophy, involuntary muscle twitching, impaired voluntary speech (dysarthria), difficulty swallowing (dysphagia), and breathing difficulties in advanced stages [1,2]. Although cognitive faculties remain functional in the initial phases, some patients may develop frontotemporal dementia. Currently, ALS is considered a fatal illness, with life expectancy ranging from 2–5 years post diagnosis. Approximately 90 to 95 percent of cases are sporadic (sALS), while 5 to 10 percent are familial (fALS), due to mutations in SOD1, TARDBP, FUS, and C9ORF72. Proteins within motor neurons accumulate abnormally, leading to motor neuron proteinopathy [3,4]. Mutations in these genes disrupt key cellular mechanisms, spanning mitochondrial function, RNA metabolism, protein turnover, DNA repair, inflammatory signaling, and intracellular transport dynamics [5]. ALS is yet to be proven definitively curable; however, medications can aid in decelerating progressive stages of the disease. ALS involves an intricate combination of environmental, molecular, and genetic factors.

Muscle tissue is known for its high regeneration potential, which is mainly provided by satellite cells—specially committed muscle stem cells that can be turned on, multiply, and distinguish into the new fibers required, even after a strong injury [6]. However, in amyotrophic lateral sclerosis (ALS), a disease that at the same time affects both neurons and muscles, this regenerative potential is significantly diminished. Manzano et al. showed that myoblasts that had the potential to be converted into myotubes and muscle fibers were definitely reduced; the myogenic process was disrupted in mice with mutations in SOD1 or in vesicle-associated membrane protein-associated protein B (VAPB) [7,8,9,10].

Muscle tissue samples from ALS patients often demonstrate signs of impaired regeneration [11]. In the past, the muscle atrophy in ALS has been attributed mainly to denervation, i.e., motor neuron loss—muscle fiber atrophy [12].

To date, there are no therapies that can fully restore muscle structure in ALS. The treatments that have been approved, like Riluzole and Edaravone (the latter only has limited approval in some areas), primarily focus on slowing down the progression of the disease instead of reversing muscle damage [13,14,15]. Recently, however, new treatments, such as the combination of sodium phenylbutyrate and taurursodiol (known as AMX0035, Relyvrio, or PB/TURSO) and tofersen have gained FDA approval. These options are showing encouraging results in delaying functional decline and possibly helping to maintain muscle function [16,17]. This review explores the complex role of skeletal muscle in ALS. It specifically focuses on the molecular and cellular defects affecting myogenesis and myoblast fusion. The aim is to draw greater research attention to this often-overlooked aspect of the disease, with the perspective of improving patients’ quality of life.

## 2. An Overview on Muscle Tissue Biology

### 2.1. Myogenesis and Regeneration of Tissue

Skeletal muscle is one of the three main types of muscle tissue; it plays a crucial role in our ability to move voluntarily, maintain posture, support our internal organs, and regulate body temperature [18]. These muscles are attached to bones, skin, or other muscles via tendons, which are made up mostly of dense collagen fibers that help to transmit the force created during muscle contractions [19,20]. The primary cellular unit of skeletal muscle is the myofiber, a large cell with multiple nuclei that forms when precursor cells called myoblasts fuse together during the early stages of development [21,22].

When skeletal muscle becomes injured, it has an impressive ability to regenerate. This healing process is mainly driven by satellite cells (SCs), a type of stem cell that resides between the muscle fiber membrane (sarcolemma) and its outer layer (basal lamina) [23]. In their inactive state, SCs are quiet; however, they spring into action when there is tissue damage. Once activated, they multiply and can either turn into new muscle fibers by fusing together or remain as stem cells to keep the pool of SCs intact [24,25].

The regeneration and development of muscle tissue are carefully controlled by a group of proteins known as myogenic regulatory factors (MRFs), which include MyoD, Myf5, myogenin, and MRF4 [26]. Pax3 serves as an upstream regulator, directly activating the transcription of both MyoD and Myf5. Myoblasts, which come from the embryonic myotome, produce bHLH proteins and multiply in response to certain growth factors. Once these growth signals are taken away, cell proliferation stops, fibronectin is secreted, and integrin receptors are expressed. The interaction between fibronectin and integrins is crucial for kickstarting myogenic differentiation. In addition, the direct contact between cells signals that they are permanently leaving the cell cycle [27,28].

**Figure 1:**
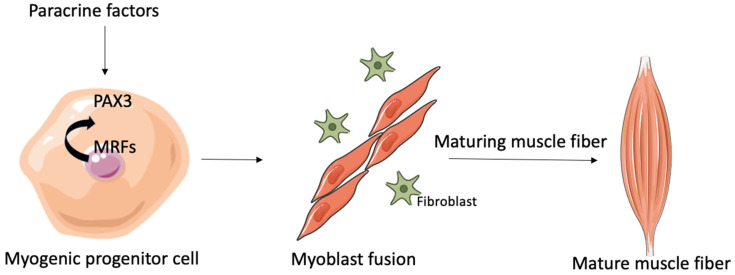
Schematic representation of myogenesis in healthy people. The image was produced using adapted templates obtained from Servier Medical Art (licensed under Creative Commons Attribution 3.0 Unported), ensuring proper attribution in accordance with the license terms. (https://smart.servier.com, accessed on 24 May 2025).

This process leads to cell fusion and the creation of myotubes. At this point, the cells lose their ability to respond to mitogens and the fused myoblasts start secreting factors that help more myoblasts join in and fuse into the developing myotube (Figure 1).

During embryonic development, structures derived from the mesoderm give rise to the body’s first muscle fibers, with additional fibers forming in waves along the initial templates [29,30]. While the mesoderm is the only germ layer that can form skeletal muscle, the specific origins and regulatory mechanisms vary depending on muscle groups such as trunk, limb, or craniofacial muscles [31]. In adult muscle, regeneration and growth are fueled by SCs, which are nestled between the basal lamina and the sarcolemma of myofibers. These cells can renew themselves; when activated, they exit their resting state and re-enter the cell cycle to produce proliferating myoblasts.

Skeletal muscle has an impressive ability to regenerate, whether under normal conditions or after an injury [32]. This remarkable capacity is mainly due to SCs, which are named for their location around mature muscle fibers. The process of muscle repair hinges on a delicate balance of pro- and anti-inflammatory signals that ultimately decide if the regeneration leads to functional muscle fibers or results in scar tissue formation [33].

Muscle regeneration occurs in two closely linked phases: degeneration and regeneration. During the degeneration phase, myofibers break down and immune cells move in. Among these, macrophages play a crucial role by clearing away cellular debris, degraded myofilaments, and damaged components of the sarcolemma. Once an injury occurs, the regenerative processes kick in; dormant SCs spring into action, entering a phase of rapid proliferation that increases the number of myogenic progenitors. This growth occurs through asymmetric cell division, where one daughter cell keeps its stem cell properties while the other starts its journey toward becoming a myoblast, which can either create new fibers or repair existing ones [34].

The regenerative response is carefully controlled by muscle-specific bHLH transcription factors such as MyoD, Myf5, myogenin, and MRF4. After muscle injury, Myf5 and MyoD are usually the first to show up in the regenerating cells, followed by myogenin and, eventually, MRF4 [35]. While both MyoD and Myf5 are key players in the early response, they have different roles; MyoD drives the final differentiation of SCs, whereas Myf5 helps maintain their ability to renew themselves [28,36].

### 2.2. Myoblast Fusion

Myoblast fusion is the fascinating process that leads to the formation of syncytial muscle fibers. This can occur in two ways: either between individual myoblasts, known as primary fusion, or between myoblasts and existing myotubes, referred to as secondary fusion. This process is crucial for the regeneration of skeletal muscle. When muscle injury occurs, it triggers SCs to spring into action and undergo asymmetric division to create new myoblasts while still preserving the stem cell pool [37].

Research into the mechanics of this process reveals that muscle cell fusion unfolds in at least three distinct phases before a fusion pore is formed [38,39]. It all kicks off when myoblasts exit the cell cycle. In conditions that promote proliferation—especially when fibroblast growth factors (FGFs) are present—myoblasts remain in an undifferentiated, proliferative state. The second phase is all about myoblasts recognizing each other and lining up in neat arrays. Finally, the third phase is where the actual membrane fusion takes place.

A wealth of studies in animal models has uncovered a variety of molecular components that are vital for the fusion process. The actin cytoskeleton is key here, playing a significant role in several stages. For instance, cytoskeletal remodeling is necessary for myoblasts to migrate toward fusion sites, and the reorganization of actin is crucial for recognition, adhesion, and vesicle trafficking [40,41]. While glycolipids and cholesterol are not as plentiful in the plasma membrane, they are still essential for influencing membrane polarity and fluidity [42]. Cholesterol, in particular, is critical for creating specialized membrane microdomains, like lipid rafts and caveolae, which help to regulate the signaling pathways related to fusion [43].

Another important group of molecules plays a role in how cells recognize and stick to each other. This process relies on specific types of integrins and cell adhesion molecules (CAMs). Membrane glycoproteins, like cadherins—particularly M-cadherin (M-cad)—are crucial for homotypic cell–cell recognition [44]. These mechanisms are active during both muscle regeneration after birth and in the development of muscles in embryos, although the related signaling pathways become more pronounced after the early stages of cell fusion. M-cad is found in both SCs and the sarcolemma. After the membranes fuse, the signaling mediated by M-cad is reduced as it moves into caveolae, which causes it to be removed from the plasma membrane and directed to the proteasome for degradation [45]. Lastly, recent research has pinpointed specific intracellular signaling pathways that control the transcription of genes essential for myoblast fusion and the remodeling of the cytoskeleton.

## 3. A Brief Overview of the Genetic Basis of Pathogenesis in ALS

Beyond its notable phenotypic variability, Amyotrophic Lateral Sclerosis (ALS) also exhibits a high degree of genetic heterogeneity (Table 1). Investigating the spectrum of genetic mutations implicated in ALS provides crucial insights into the molecular and cellular pathways underlying the disease, thereby deepening our understanding of its pathogenesis.

About thirty years ago, researchers identified SOD1 (superoxide dismutase 1) as the first gene linked to ALS, especially in familial cases, where it is responsible for roughly 20% of fALS instances. Some of the most common SOD1 mutations include G93A, A4V, H46R, and D90A. Most of these mutations are passed down in an autosomal dominant way; however, D90A is somewhat of an outlier, as it can also be inherited in a recessive manner, particularly in Scandinavian populations [46,47].

Finding SOD1 was a game-changer, paving the way for the creation of disease models that have been crucial in revealing several important mechanisms behind ALS. When mutant SOD1 proteins accumulate, they create a toxic gain-of-function that leads to the degeneration of motor neurons, which is a key feature of the disease [48,49]. A characteristic feature of ALS motor neurons is the accumulation of intracellular inclusions that are positive for both SOD1 and ubiquitin [50]. Mutant SOD1 tends to accumulate in the cytoplasm, forming aggregates that disrupt neuronal function by sequestering vital proteins necessary for cell survival. These aggregates interfere with the ubiquitin–proteasome system (UPS), deplete molecular chaperones, compromise mitochondrial integrity, and impair both cytoskeletal dynamics and axonal transport, ultimately leading to neuronal death [51].

In 2006, researchers made significant strides in understanding ALS by identifying TDP-43 as a key pathological protein found in the cytoplasmic inclusions of both sporadic ALS (sALS) and familial ALS cases that are not linked to SOD1 mutations (non-SOD1 fALS) [52]. TDP-43 is a nucleic acid-binding protein that regulates transcription and coordinates several aspects of RNA metabolism, including splicing and transport, and it is recognized as a major player in the development of ALS [53]. Research has indicated that mutations in TARDBP can lead to neuronal damage through toxic gain-of-function effects caused by dysfunctional TDP-43. The majority of disease-causing mutations in TARDBP cluster within the glycine-rich C-terminal domain, which is essential for controlling nucleocytoplasmic shuttling, aggregation tendencies, and protein interaction networks [54]. Beyond ubiquitination, abnormal phosphorylation of TDP-43 is a critical event, promoting the formation of pathogenic protein aggregates; ALS patient samples reveal pronounced phosphorylation at the C-terminus, which enhances aggregation propensity [55].

Subsequent breakthroughs in ALS genetics identified mutations in the FUS/TLS gene, another RNA/DNA-binding protein implicated in familial ALS. FUS plays a pivotal role in mRNA transport to dendrites and supports synaptic plasticity following glutamate receptor stimulation [56,57]. Pathogenic variants in FUS predominantly affect the nuclear localization signal (NLS) domain within the C-terminal region, disrupting the proper cellular distribution of the protein—a mechanism shared with TDP-43 [58,59].

In 2011, researchers made a groundbreaking discovery when they found a pathogenic expansion of GGGGCC (G4C2) hexanucleotide repeats in the first intron of the C9ORF72 gene. This finding established it as the main genetic culprit behind both ALS and frontotemporal dementia (FTD) [60]. Three key pathogenic mechanisms have been suggested: a loss-of-function effect due to decreased levels of C9ORF72 mRNA and protein; RNA-mediated toxicity caused by the bidirectional transcription of repeat-containing RNAs, which trap vital RNA-binding proteins (RBPs) into RNA foci; and toxicity linked to the production of repetitive dipeptide repeat proteins through unconventional translation of these expanded transcripts [61].

Additionally, disruptions in various downstream cellular pathways, like nucleocytoplasmic trafficking and autophagy, have been associated with the disease’s progression. It is particularly noteworthy that patients with C9ORF72 expansions show significant dysfunction in the ubiquitin–proteasome system (UPS), with ubiquitin-positive neuronal inclusions serving as a key pathological feature [62].

In addition to the genes classically associated with motor neuron pathology, an increasing number of studies have reported dysregulation of muscle-intrinsic genes in ALS. Among the most affected are the myosin heavy chain (MYH) isoforms, whose altered expression may reflect an imbalance in fiber-type composition and a shift toward a more glycolytic profile [63]. This fiber-type switching can reduce endurance capacity and disrupt force generation. Furthermore, key structural and cytoskeletal components—including desmin, dystrophin, and titin—are often found to be mis-expressed or structurally altered in ALS muscle biopsies [64]. These proteins are essential for sarcomeric alignment, contractile force transmission, and muscle fiber integrity. Their dysfunction may contribute not only to atrophy but also to mechanical instability and increased susceptibility to injury. These findings reinforce the concept that skeletal muscle in ALS is not merely degenerating in response to denervation; it may also be undergoing primary, cell-autonomous changes at the transcriptional and structural level.

## 4. Muscle Pathophysiology in ALS: Mechanisms and Implications

In this pathology, the progressive loss of muscle mass stems largely from the interruption of nerve signals caused by motor neuron degeneration. This deterioration begins with the loss of lower motor neurons, which compromises the function of neuromuscular junctions (NMJs)—the critical interfaces that relay impulses from nerves to muscle fibers. As these junctions break down, muscle fibers are deprived of the stimulation needed to maintain normal function. The absence of neural input leads to reduced contractile activity, which triggers a gradual decline in muscle fiber size (atrophy) and, eventually, the loss of muscle fibers altogether [65].

Various cellular processes are involved in driving muscle atrophy in ALS (Table 2).

### 4.1. Mitochondrial Dysfunction

Mitochondrial dysfunction is important process of muscle involvement in ALS [66]. Mitochondria in ALS-affected muscles exhibit structural abnormalities, such as swollen and fragmented cristae, which impair their function. These mitochondrial defects reduce ATP synthesis while increasing ROS production [67]. The resulting bioenergetic deficits and oxidative stress further damage muscle cells and contribute to the progression of muscle atrophy [68]. Mitochondria are the powerhouses of the cell; their dysfunction in ALS leads to an energy crisis within muscle cells, compromising their viability and function. Moreover, the accumulation of ROS causes oxidative damage to proteins, lipids, and DNA, further exacerbating muscle cell death [66,69]. This is a cellular mechanism aimed at counteracting ROS and the cellular damage it produces. A study by Vielhaber and colleagues demonstrated that Mn-SOD deficiency in human muscle tissue appears to be a process involved in sporadic ALS; this was confirmed in recent years by Yan at al. [70,71]. These results were again later confirmed by subsequent investigations, including one by Xiao et al., demonstrating that reactive oxygen species (ROS) accumulation and oxidative stress were present in the skeletal muscles of both ALS patients and SOD1-G93A transgenic mice, supporting the idea that these mechanisms are relevant across both clinical and experimental models of ALS [72]. These animals, in particular, exhibited overexpression of cyclophilin D (CypD), which contributed to ROS production [72,73]. Excessive ROS production has also been reported to cause mitochondrial DNA (mtDNA) damage, leading to defects in the respiratory chain within skeletal muscle mitochondria of fALS patients [66,70], further impairing ATP production and mitochondrial function. Protein aggregation—a hallmark of ALS also observed in muscle tissue—has been hypothesized, primarily from preclinical models, to worsen oxidative stress. This remains speculative, as confirmation in human muscle is still limited.

This occurs because maintaining correct protein folding is energy-intensive, thereby overloading the mitochondrial oxidative phosphorylation system and amplifying ROS generation [69].

### 4.2. Protein Degradation

Another important process involved in muscle pathology in ALS is the ubiquitin–proteasome system (UPS), which is responsible for the disposal of damaged proteins [74]. In ALS, there is increased expression of muscle-specific E3 ligases, such as MuRF1 and atrogin-1/MAFbx, which target muscle proteins for degradation [75]. This enhanced proteolytic activity significantly contributes to muscle mass loss and plays a major role in the development of severe muscle atrophy [74]. Additionally, the autophagy–lysosome pathway (ALP) is also altered in ALS [76]. Autophagy, the process through which cells degrade and recycle their own components, becomes overactive in ALS-affected muscles, leading to excessive protein breakdown. Evidence from transgenic mouse models suggests that dysregulated autophagy might directly damage muscle tissue, beyond its role in neurons [77]. However, the extent to which this contributes to ALS muscle degeneration in patients remains unclear.

A third key process is apoptosis. In ALS, there is an imbalance between pro-apoptotic and anti-apoptotic proteins, with an increase in Bax and a reduction in Bcl-2, which promotes muscle fiber death. This apoptotic cascade is triggered by various stress signals, including mitochondrial dysfunction and oxidative stress, ultimately leading to the orderly dismantling of muscle cells [69,78].

### 4.3. Muscle Denervation and Reinnervation

An additional altered process involved in muscle pathology in ALS concerns the interaction between denervation and reinnervation. This is a distinctive feature of ALS, reflecting the degenerative nature of the disease. In the early stages, surviving motor neurons attempt to compensate for neuronal loss by extending axons to reestablish contact with denervated muscle fibers. This adaptive reinnervation is only temporary in preserving muscle function [79]. However, as the disease progresses, the capacity for reinnervation gradually declines, ultimately leading to widespread and irreversible muscle atrophy [80]. This process also involves a remodeling of motor units; however, the relentless neurodegeneration eventually overcomes these compensatory mechanisms, contributing to the progressive deterioration of muscle function [81]. Another well-documented feature of ALS is the shift in muscle fiber-type composition. The disease primarily affects fast-twitch fibers (type II), which are more vulnerable to denervation [82]. Subsequently, slow-twitch fibers (type I)—initially preserved—also begin to atrophy as the disease progresses [82]. This fiber-type transition alters the biomechanical properties of muscle, compromising both strength and endurance. Fast-twitch fibers are crucial for quick, powerful movements, while slow-twitch fibers support sustained activity over time. The selective vulnerability of fast-twitch fibers disrupts this balance, contributing to the muscle weakness and fatigue commonly observed in ALS patients [83].

Although the mechanisms underlying this selectivity are not yet fully understood, several hypotheses have been proposed. One explanation involves oxidative stress; fast-twitch fibers, which rely mainly on anaerobic glycolysis, are more prone to accumulation of reactive oxygen species (ROS). Compared to slow-twitch fibers, fast-twitch fibers suffer more severe oxidative damage, as demonstrated by numerous studies [84,85].

Another proposed contributing factor is the greater sensitivity of fast-twitch fibers to apoptotic signals, making them more likely to undergo programmed cell death in ALS [82]. Additionally, the type of motor neuron innervating the muscle fiber plays a role in susceptibility; fast-twitch fibers are typically innervated by motor neurons that are more prone to degeneration in ALS [86].

### 4.4. Neuromuscular Junction (NMJ)

The last important point in this section is the neuromuscular junction (NMJ). NMJ is a highly organized structure that enables communication between motor neurons (MNs) and muscle fibers, triggering muscle contraction [87]. This connection is supported not only by neurons and myofibers but also by specialized glial cells, such as terminal Schwann cells (TSCs) and kranocytes, which are essential for maintaining NMJ integrity and promoting repair after injury [88].

In ALS, NMJ degeneration emerges as a key event in the disease’s development, primarily driven by axon degeneration. As motor neurons deteriorate, NMJs become denervated, reducing their ability to regenerate and weakening the chances of successful reinnervation [89]. Notably, the suppression of mutant TDP-43 has been shown to encourage axonal regeneration in experimental models, suggesting a degree of plasticity in early disease stages. Crucially, NMJ impairment can begin before motor symptoms become evident [90]. Alterations in the presynaptic compartment, particularly in TSCs, are significant. In ALS, TSCs exhibit abnormal morphology and reduced regenerative capacity. Experimental studies using SOD1-G37R mice show that these defects contribute to the disorganization of motor units and precede axonal loss [89]. Additionally, NMJs located farther from the spinal cord are more vulnerable than those in proximal regions. Research in SOD1-G93A models indicates that even before clinical onset, these Schwann cells fail to properly mediate NMJ repair following nerve injury [91]. At the postsynaptic level, disruptions in pathways that regulate nicotinic acetylcholine receptor (nAChR) clustering can compromise NMJ stability. Mutant proteins, such as FUS, often found in ALS, can destabilize these synaptic sites. Furthermore, SCs secrete factors that guide axonal navigation and support NMJ regeneration, especially under stress or injury conditions [92]. Among these, SEMA3A, mainly produced by muscle tissue, is crucial for motor neuron regrowth and synaptic reformation [89].

Key molecular mediators, such as agrin, coordinate synaptic stability by activating LRP4-MuSK signaling, which is necessary for maintaining nAChR clustering. FUS may interfere with this system through its interaction with transcriptional regulators. The importance of SCs has been confirmed by studies showing that their absence results in failed NMJ regeneration and accelerated synaptic degeneration [93].

In addition, studies have reported acetylcholine receptor (AChR) fragmentation at the NMJ in ALS models, reflecting postsynaptic destabilization. Fragmented or misclustered AChRs reduce synaptic transmission efficiency and contribute to functional disconnection. Electrophysiological analyses, including compound muscle action potential (CMAP) recordings, further support this by showing increased latency and reduced amplitude, consistent with impaired nerve-to-muscle signal conduction [94].

In ALS, muscle pathology arises from a complex interplay of mechanisms including denervation, mitochondrial dysfunction, altered proteostasis, and impaired regeneration. These disruptions converge to accelerate muscle atrophy and functional decline. The neuromuscular junction plays a pivotal role in disease onset and progression. Preserving NMJ integrity and muscle homeostasis may represent a promising therapeutic strategy.

## 5. Altered Regenerative Response of Skeletal Muscle in ALS

In the previous sections, we examined both the biology of muscle tissue and the various molecular pathways that can lead to muscle damage in ALS, ultimately resulting in the formation of fibers with altered morphology due to defective regenerative mechanisms. Section 5 will explore, in more detail, the complex biology of muscle regeneration in ALS-affected tissue.

**Figure 2:**
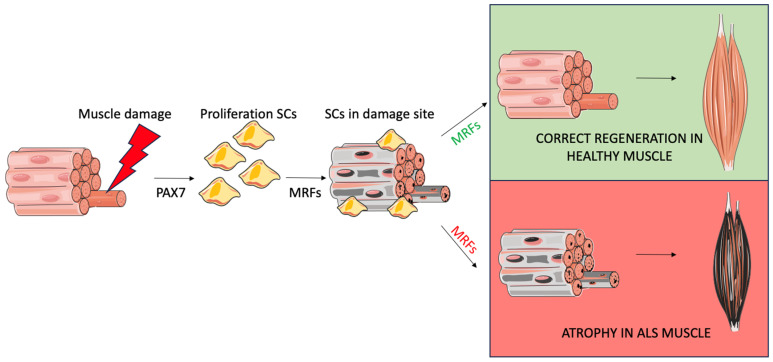
Illustration of the muscle regeneration process that occurs after an injury in healthy muscle compared with ALS muscle. The image was produced using adapted templates obtained from Servier Medical Art (licensed under Creative Commons Attribution 3.0 Unported), ensuring proper attribution in accordance with the license terms. (https://smart.servier.com, accessed on 24 May 2025).

One of the most important features of skeletal muscle, as previously mentioned, is its ability to repair and regenerate. Muscle regeneration relies mainly on SCs, which reside between the basal lamina and the sarcolemma and serve as stem cells activated in response to damage. These cells are highly resilient and can remain in a quiescent state until activated by physiological stimuli such as tissue injury or exercise-induced damage [95,96] (Figure 2).

In healthy adult muscle, quiescent SCs express Pax7, a transcription factor essential for their survival, maintenance, and regenerative potential [96,97]. Upon activation, SCs re-enter the cell cycle and proliferate, initially co-expressing MyoD, an early myogenic marker. As differentiation progresses, SCs upregulate myogenin (Myog), after which myoblasts fuse to form myotubes, thereby contributing to the regeneration of damaged muscle fibers [35,97]. As previously discussed in the earlier chapters, this entire process is regulated by a family of transcription factors known as myogenic regulatory factors (MRFs), including MyoD, Myf5, Myog, and MRF4. These proteins act through their basic helix–loop–helix (bHLH) domain, binding to conserved DNA motifs called E-boxes (CANNTG) that are found in the regulatory regions of both muscle-specific and non-muscle-specific genes [35]. MRFs can form homo- and heterodimers, allowing for the fine-tuned regulation of gene expression during muscle regeneration.

Studies in SOD1-G93A mouse models of ALS have shown elevated mRNA levels of Pax7 and myogenic regulatory factors (MRFs) during the late stages of the disease. Interestingly, these increases are not mirrored at the protein level, suggesting a possible disconnect between transcriptional activation and effective muscle regeneration [98]. One possible interpretation is that this reflects a compensatory attempt by the muscle to activate SCs in response to ongoing denervation. However, the lack of protein accumulation may also indicate intrinsic defects in post-transcriptional regulation or protein turnover. Whether this dysregulation represents a failed adaptive response or a primary pathogenic mechanism remains unresolved. Clarifying this distinction is essential to determine if targeting satellite cell pathways could modify disease progression or merely support damaged tissue.

Moreover, it has been observed that different muscle-fiber types show varying degrees of sensitivity to SOD1-G93A-induced toxicity. Studies suggest that the differential response may stem from distinct metabolic pathways or stress responses in fast- versus slow-twitch fibers [99,100,101].

Histological analysis of muscle biopsies from ALS patients has revealed an increase in euchromatin within SCs, indicating transcriptional activation, although the total SC count remains unchanged [101]. Molecular studies have confirmed this through the reduced expression of Pax7 and Myf5, along with the presence of Pax7+ cells lacking MyoD expression, suggesting that while SCs are activated, they fail to complete the myogenic program [102,103].

These in vivo findings are further supported by in vitro studies, which have demonstrated impaired differentiation, abnormal myotube morphology, and reduced expression of myosin heavy-chain (MHC) isoforms—including fast, slow, and neonatal variants—as well as lower Myog protein levels [7,12]. Additionally, despite the total number of Pax7+ cells being similar to that of controls, ALS muscle biopsies display very few nuclei that are Pax7-positive but Myog-negative, suggesting a senescent state of SCs [12].

In this context, emerging hypotheses point to the role of altered phase-separation dynamics in satellite cells as a potential contributor to regenerative failure in ALS muscle. RNA-binding proteins, such as TDP-43 and FUS, which are involved in ALS pathogenesis, normally undergo liquid–liquid phase separation to form functional ribonucleoprotein granules. When mutated or misregulated, they may form persistent or aberrant aggregates, disrupting mRNA metabolism within SCs. This could impair key post-transcriptional processes during SC activation and differentiation, contributing to regenerative inefficiency. While this hypothesis remains to be experimentally validated in muscle tissue, it highlights a possible link between protein homeostasis and defective myogenesis in ALS [104].

Altogether, these observations support the idea that SC dysfunction contributes to impaired muscle regeneration in ALS. However, it remains uncertain whether these alterations are a consequence of motor neuron degeneration or whether they play a more active, causative role in disease progression. This ambiguity underscores the need for mechanistic studies capable of dissecting muscle-autonomous versus neuron-dependent pathways in ALS-related atrophy.

Understanding these alterations in muscle stem cells and the regeneration process is of paramount importance, as it underscores the role of muscle, not just as a passive target, but as an active player in ALS pathogenesis—and potentially as a therapeutic target for restoring regenerative capacity and preserving muscle function.

## 6. Physical Activity as a Therapy for ALS Muscle

**Figure 3:**
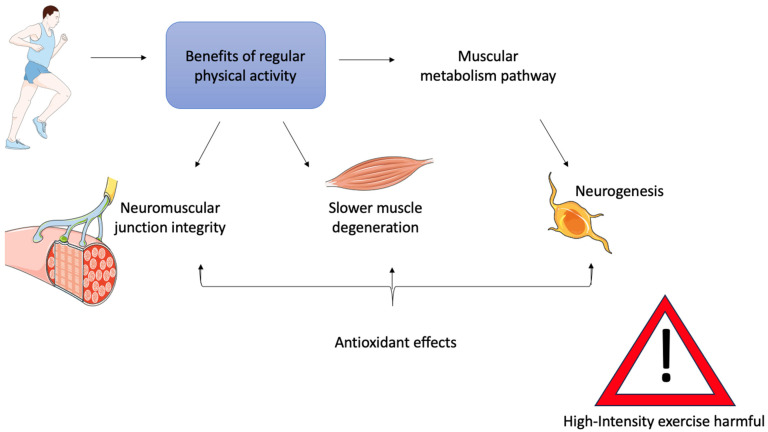
Summary representation of possible benefits of regular physical activity as a therapy for ALS patients. The image was produced using adapted templates obtained from Servier Medical Art (licensed under Creative Commons Attribution 3.0 Unported), ensuring proper attribution in accordance with the license terms. (https://smart.servier.com, accessed on 24 May 2025).

Regular physical activity offers clear benefits to patients affected by ALS (Figure 3) [105,106].

Consequently, it is important to consider whether exercise therapy may help to slow muscle degeneration and preserve neuromuscular junction (NMJ) integrity in patients. Researchers are currently evaluating this approach through various studies, as physical activity is believed to activate metabolic pathways in muscle, increase glucose uptake, and promote healthy muscle regeneration [107]. In addition, physical activity has antioxidant effects, and can stimulate mitochondrial biogenesis in muscle as well as neurogenesis, thereby improving the relationship between muscle and motor neurons [108,109,110]. Indeed, several studies have observed higher levels of circulating antioxidants in MRL/MpJ mice, a mouse model widely used to investigate molecular and cellular mechanisms of tissue regeneration due to their high repair capacity. Moreover, it was found that deletion of the Sod1 gene impaired the myogenic capacity of these mice, suggesting a link between genetic mutations and the regulation of muscle regeneration [111].

However, it is also important to emphasize that physical activity, if not properly regulated, can be harmful. It has been shown that excessive levels of the same physical stimulus can have negative or even toxic effects on muscle [112].

Properly dosed and consistently performed moderate-intensity physical therapy is known to reduce the cellular damage caused by inflammatory processes induced by ROS [113,114], and to have a protective effect against the mitochondrial dysfunction associated with aging and commonly seen in ALS, as discussed in previous chapters [115,116]. In contrast, continuous high-intensity training has been shown to have the opposite effect [117].

Promising studies have also been published on the benefits of aquatic physical activity. Notably, swimming was found to extend the lifespan of SOD1-G93A mutant mice more effectively than running [118,119]. This is thought to be because swimming engages fast motor units, while running primarily activates slow motor units [119,120].

One study on SOD1-G93A mouse models investigated the effects of swimming on the BDNF/TrkB signaling pathway [121]. Brain-Derived Neurotrophic Factor (BDNF) is a protein secreted during muscle contraction that can act as both a neuroprotective and neurotoxic agent. In these mice, neuronal hyperexcitability and the resulting muscle contractions caused excessive BDNF secretion, which likely contributed to neurodegeneration by increasing glutamate toxicity [121].

Studies in ALS patients undergoing standard therapy and those also participating in exercise programs have reported some beneficial effects on muscle condition and functional capacity [122,123]. However, these findings are not always consistent across trials. Several clinical studies have been limited by small sample sizes, short follow-up durations, and heterogeneity in patient baseline characteristics and exercise protocols [124]. While some trials observed modest improvements in strength or quality of life, others failed to show significant clinical benefits. Therefore, further large-scale and well-controlled studies are necessary to clarify the true therapeutic potential of exercise in ALS.

Emerging pharmacological treatments, such as AMX0035 and Tofersen, have demonstrated some disease-modifying potential in ALS. AMX0035 has shown a modest slowing of functional decline in ALSFRS-R scores, which include elements related to motor and muscular performance [125]. Tofersen, an antisense oligonucleotide targeting SOD1, has shown promise in reducing neurofilament levels and has been associated with a stabilization or slower decline in muscle strength in early-stage SOD1-ALS patients [126]. Both compounds have been recently approved by the FDA under conditions of accelerated approval. However, direct evidence of muscle-specific outcomes—such as improvements in muscle histology, fiber integrity, or muscle-based biomarkers—is still scarce [127]. Further studies are critically needed to determine whether these treatments provide direct benefit to skeletal muscle or primarily act through neuroprotective mechanisms.

## 7. Conclusions

Amyotrophic lateral sclerosis (ALS) has long been seen as a condition mainly caused by the degeneration of motor neurons. However, this review brings to light a growing body of evidence indicating that skeletal muscle is not just a passive victim of neurodegeneration; it actively participates in the disease process. Muscle fibers experience significant structural and metabolic changes; their ability to regenerate is greatly hindered by issues with satellite cells, disrupted myogenic signaling, and problematic post-transcriptional mechanisms.

The imbalance of crucial myogenic regulators, like Pax7, MyoD, Myf5, and Myog, along with unusual responses from various muscle fiber types—indicates that muscle degeneration in ALS is more than just a result of denervation; it is a complex, intrinsic process. These findings highlight the importance of viewing muscle as a key player in the development of ALS.

Moreover, the positive impacts of physical exercise on muscle adaptability, the stability of neuromuscular junctions, and metabolic resilience open up exciting new therapeutic possibilities. Exercise-based treatments, paired with molecular strategies aimed at enhancing myogenesis and satellite cell function, could potentially slow down disease progression and enhance quality of life.

Looking ahead, research should focus on understanding the muscle’s role as a modifiable factor in the disease, investigate muscle-centered biomarkers, and design clinical trials that take peripheral tissues into account when assessing ALS progression and treatment responses. Reframing ALS as a neuromuscular disease rather than just a neurodegenerative one—could be a vital step toward developing more comprehensive and effective therapies.

To advance this perspective, future research should prioritize the identification of muscle-derived biomarkers that reflect disease onset and progression, allowing for earlier and more precise monitoring of ALS beyond the central nervous system. In parallel, therapeutic strategies aimed at preserving or restoring satellite cell function—through gene modulation, metabolic reprogramming, or anti-inflammatory approaches—should be further developed. Bridging the gap between preclinical findings and clinical application remains essential to validate the muscle’s role as both a target and a source of prognostic information in ALS.

## Figures and Tables

**Table 1:** Genetic mutations and pathological mechanisms involved in ALS.

Gene	Common Mutations	Main Pathological Mechanism	Clinical Features
**SOD1**	G93A, A4V, H46R, D90A (recessive in Scandinavian population)	- Toxic protein aggregation - UPS dysfunction - Oxidative stress - Mitochondrial and axonal transport damage	- Typical of fALS (~20%) - Ubiquitin-positive aggregates in motor neurons (MNs)
**TARDBP (TDP-43)**	Mutations in the glycine-rich C-terminal region	- Impaired nucleo-cytoplasmic trafficking - Phosphorylated protein aggregates - RNA/transcriptional dysfunction	- Found in both sALS and non-SOD1 fALS - TDP-43/ubiquitin-positive aggregates in MNs
**FUS/TLS**	Mutations in C-terminal region of NLS	- Impaired mRNA transport - Cytoplasmic aggregation - Disruption of synaptic plasticity regulation	- fALS - Phenotypic and age-of-onset variability - FUS-positive cytoplasmic inclusions
**C9ORF72**	GGGGCC (G4C2) intronic hexanucleotide expansion	- Loss of C9ORF72 function - RNA-mediated toxicity via repeat-containing RNAs - Toxic dipeptides (RAN translation)	- Most common genetic cause of ALS and FTD - Ubiquitin-positive neuronal inclusions

**Table 2:** Summary of cellular and molecular mechanisms involved in muscle pathophysiology in ALS.

Mechanism	Physiological Role	Alterations in ALS	Consequences on Muscle
**Neuromuscular innervation**	Signal transmission between motor neurons and muscle fibers	Motor neuron degeneration and progressive loss of neuromuscular junctions (NMJs)	Denervation, muscle atrophy
**Mitochondria**	ATP production, redox homeostasis	Structural disorganization, increased ROS, mitochondrial DNA damage	Energy crisis, oxidative stress
**Proteostasis (UPS)**	Degradation of damaged or misfolded proteins	Accumulation of toxic protein aggregates (e.g., mutant SOD1), UPS dysfunction	Cellular toxicity, pathological inclusions
**Autophagy**	Clearance of damaged organelles and protein aggregates	Dysregulated activation or chronic stimulation	Impaired turnover, buildup of dysfunctional components
**Local inflammation**	Controlled immune response to injury	Chronic activation of microglia and astrocytes, increased TNF-α and IL-1β secretion	Hostile environment, inhibition of regeneration
**Muscle fiber structure**	Contraction, adaptation to mechanical stimuli	Reduction in cross-sectional area, selective loss of fast-twitch fibers	Progressive weakness, altered morphology
**NMJ and synaptic signaling**	Stabilization of acetylcholine receptors and nerve-muscle communication	Disruption of pre/post-synaptic elements, loss of nAChR clustering	NMJ instability, functional impairment

## Data Availability

No new data were created or analyzed in this study.
